# Layperson and Healthcare Professional Experiences of Medical Misinformation About Masks on Social Media During the Covid-19 Pandemic: A Thematic Framework Analysis

**DOI:** 10.1007/s10728-025-00546-x

**Published:** 2025-11-03

**Authors:** Daniel Robinson, Steven Agius, Alistair Warren

**Affiliations:** School of Medicine, University of Nottingham, Nottingham, UK

**Keywords:** Medical misinformation, Face masks, Social media, Public health education, Covid-19

## Abstract

This study aimed to compare how healthcare professionals and laypeople from the UK engaged with and confronted misinformation about masks on social media and some of the barriers encountered, applying connectivism as a theoretical lens to interpret findings. Using thematic framework analysis, 12 in-depth interviews were conducted with participants consisting of seven healthcare professionals and five laypeople who actively use the social media website Twitter (now known as X). Interviews were recorded, transcribed verbatim and analysed iteratively using constant comparison and line-by-line coding. After coding of the first few interviews a working analytical framework was developed by comparing codes and grouping them together into categories. This was later applied to the subsequent interview transcripts. Analysis revealed six key categories: social capital (e.g., celebrity status), emotion (e.g., fear, anger, and burnout), culture (e.g., politics and toxic social media culture), social media dynamics (e.g., information overload), communication (e.g., managing cognitive load), and belief (e.g., beliefs being fixed and difficult to change). Our study suggests that healthcare professionals must actively engage with the public on social media by providing clear, evidence-based information, correcting false claims, and fostering trust through transparent communication. Interpreting our findings through a connectivist perspective highlights how digital networks shape trust and learning in online health discourse. Wider society, including tech companies and policymakers, should implement stronger fact-checking mechanisms, promote digital literacy, and support regulations that curb the spread of harmful health misinformation.

## Introduction

 Covid-19 devastated societies around the world and highlighted a multitude of issues, including medical misinformation. The pandemic led to medical practitioners and scientists having to contend with misinformation at all levels. Media coverage in the United Kingdom (UK) highlighted misinformation related to the Covid-19, for example videos of families attempting to remove ventilated relatives from hospitals due to their belief that Covid-19 did not exist [22]. Negative sentiment towards various preventative measures, including mask wearing, was shared widely on social media [27, 50]. Galloti et al. found that measurable waves of potentially unreliable information preceded the rise of Covid-19 infections, suggesting that misinformation poses a serious threat to public health [17]. Similarly, Al-Ramahi et al. reported a strong positive correlation between an increase in volume of anti-mask tweets and new Covid-19 cases 9 days later [1]. Thus, understanding how to contend with this issue is of paramount importance to public health.

Although this research refers to misinformation during the period of the Covid-19 pandemic, it is important to note that such misinformation is widespread in many areas such as cancer treatment [5] and has been reported recently in the case of Paloma Shemirani [46, 47].

Manipulating emotion is a means through which to influence public opinion and drive societal change. This idea has a long history, for example advertising campaigns targeting women in the early 20th century describing cigarettes as “torches of freedom”, or a symbol of liberation against patriarchal norms of the time [2]. Conspiracy groups exploit negative emotions such as fear in order to promote conspiracist narratives [7, 21, 40]. Similarly, during the pandemic anti-vaccine groups have been shown to frame their messaging as an advocacy campaign to protect children [24]. This may be in line with the theory of care ethics, which argues that moral actions centre on interpersonal relationships.

In the modern world, a culture of mistrust in experts has developed within certain groups. Epistemic labour refers to the labour time required to acquire and use knowledge. A healthcare practitioner’s epistemic labour invested in understanding a healthcare phenomenon such as Covid-19 should arguably put them in a position of trust. However, during Covid-19 anti-intellectualism was prevalent and has been shown to be associated with reduced compliance with mask-wearing [35]. This may be considered an atypical type of epistemic injustice, which is when a person’s knowledge is unfairly dismissed because of their social identity [15], where a healthcare professional’s knowledge is dismissed simply because they are an ‘expert’. This leads to people finding information from other less reputable sources.

Celebrities or influencers are one such source. Para-social interaction is a theory that has been used in social media research to study user behaviour in relation to branding and purchasing intentions of people online [39]. For example, Colliander et al. found that blogs were more effective at promoting publicity than traditional online media such as magazines because consumers form connections with their blogger ‘friends’ which leads them to forming ‘friendships’ with the brands they promote [11]. These online relationships lead to increased trust in the influencer as a source and, in marketing research, increase purchase intentions [9, 53]. A similar phenomenon was reported by Lockyer et al. whose participants acknowledged that if conspiratorial ideas about Covid-19 were shared by people they trusted they were difficult to ignore [30]. This suggests that parasocial relationships and relational trust may be important in the development of health beliefs.

The social media environment is oversaturated with information, which impacts the capacity for learning. Cognitive load, a theory first described by Sweller et al. [48], describes how the information processing load induced by learning tasks can affect learner’s ability to process information. The relevance of cognitive load to misinformation during Covid-19 has been reported by three studies [28, 31, 38]. Therefore, cognitive load may be a significant barrier to counteracting misinformation about masks.

Effective communication is one way to manage cognitive load. Zhang et al. found that inconsistencies in messaging fostered confusion and led to some mistrust in healthcare professionals [55]. They also found that there were deficits in health information related to the use, effectiveness and benefits of mask wearing. In terms of communication strategies, a number of studies about health promotion during Covid-19 have supported the use of infographics to counteract misinformation [14, 51]. This may be one way in which cognitive load can be addressed, providing clear and easily digestible information about mask use.

Counteracting health misinformation is a complex challenge with many barriers. Bautista et al. identified barriers to healthcare professionals engaging online including: intrapersonal barriers such as lack of positive outcome and lack of time; interpersonal barriers for example harassment and bullying, and difficulty having meaningful conversations on the web; and institutional barriers such as lack of organisational support and lack of social media training [3]. They further found that perceived organisational support made people more likely to have a positive attitude towards, feel social pressure to, and be confident in correcting misinformation [4].

Overall, public health education and misinformation can be seen as two poles of a learning process, and answers to misinformation could therefore be sought through pedagogical theory. Connectivism is the theory that epistemologically, knowledge is formed through the process of a learner connecting to and feeding information into a learning community, described as a node, which is in turn part of a larger network [44]. The response of individuals to misinformation or accurate information about masks on social media therefore could fit into this model.

The aim of the present study was to compare how healthcare professionals and laypeople from the UK engaged with and confronted misinformation about masks on social media and some of the barriers encountered. The study was guided by the following research questions:

1. How did healthcare professionals and laypeople in the UK experience, interpret and respond to misinformation about mask-wearing on social media during the Covid-19 pandemic?
2. What social, emotional and communicative factors shaped their engagement with online misinformation?
3. How can these experiences be understood through the theoretical lens of connectivism, which conceptualises learning and knowledge as emergent from networks of connection and trust?

## Conceptual Framework and Methodology

The theoretical lens of connectivism was used to guide decisions about the methodology of this study. According to Siemens, the starting point of connectivism is the individual, whose knowledge is comprised of a network, which then feeds into organisations and institutions, and subsequently feeds back into the network continuing to provide learning to individuals [44]. Thus, methodological frameworks which would align most with a connectivist viewpoint would be those where theories are generated through individual participants standpoints.

With this in mind, this study was conducted using the analytical framework method to analyse data from individual interviews [43]. This was chosen as it is a systematic methodology that is heavily based in and driven by the accounts of people that it is about, enabling comprehensive analysis of all the material collected [43].

Ethical approval was granted by the University of Nottingham Faculty of Medicine and Health Sciences Research Ethics Committee – Ethics reference number: FMHS 230–0321. All participants gave informed consent for involvement in the study and for publication of anonymised data.

Data were collected through individual interviews. Interviews were performed via Microsoft Teams over a period of six months from January to June 2022 and were between 30 min and 1 h in duration. Interviews were recorded and transcribed verbatim by hand. After participants had taken part in interviews, they were sent an information sheet outlining the evidence for the efficacy and safety of masking.

Participants were initially recruited through a Twitter account created specifically for the study, asking friends and associates of the author to share information about the study with their followers, using snowball sampling. In addition, the recruitment account engaged with posts about masks on social media to further spread information about the study. To attempt to recruit a diverse group of participants this included engaging with both pro- and anti-mask posts, as well as large social media accounts across the political spectrum. Unfortunately, recruitment through this method was limited. The details of the study were, therefore, further shared through local presentations, the University of Nottingham Academic Medical Education Blog, and through the social media accounts of a medical education organisation (Junior Association for the Study of Medical Education). The exclusion criteria are included in Table 1 below. Participants were not offered any incentives to take part in the study.

**Table 1:** Exclusion criteria

Exclusion Criteria
Participant living outside the UK
Age < 18
Unable to give informed consent e.g., learning disability affecting capacity to make decisions
Inactive on social media (defined as using social media less than once per week)
Twitter account started after 01/01/2020
Non-English speaker

Twelve participants were recruited and interviewed by the lead author in 1–1 interviews including seven healthcare professionals and five laypeople. Sociodemographic details of participants are included in Tables 2 and 3 including age, ethnicity, gender, job role, educational level, frequency of social media use, and political party voted for in the last election. Unfortunately, there were no participants who held right-wing political beliefs or who were strongly anti-mask, despite attempts to reach people with these views.

After familiarisation, the data were independently coded by the first and last authors using line-by-line coding. To mitigate individual bias and subjectivity the codes were then discussed between all authors to agree the final codes. After coding of the first few interviews, a working analytical framework was developed by comparing codes and grouping them together into categories following discussion between all authors. This was later applied to the subsequent interview transcripts. After 12 interviews a point was reached where new data would not contribute further to the research question and therefore data saturation was felt to have been reached. Once interviews had been completed data was then charted into framework matrices (Appendix 1 and 2).

**Table 2:** Demographic details of layperson participants

Participant number	Age	Ethnicity	Gender	Employed/unemployed	Job role	Educational level	Social media use	Political party voted for in the last election
A1	22-30	White	Cis-male	Employed	Project manager	Undergraduate Degree or above	Once per day	Labour
A2	31-40	White	Cis-male	Employed	Storyliner at popular UK television show	Undergraduate degree or above	Once per day	Labour
A3	41-50	White	Cis-female	No formal employment	Caring responsibilities, and homeschooled children during the pandemic	GCSE	Once per day	Labour
A4	22-30	Mixed	Cis-male	No formal employment	N/a	Undergraduate Degree or above	Once per day	Labour
A5	22-30	White	Non-binary	Employed	Freelance Radio Producer	Undergraduate Degree or above	Once per day	Labour

**Table 3:** Demographic details of healthcare professional participants

Participant Number	Age	Ethnicity	Gender	Role	Educational level	Social media use	Political party voted for at the last election
B1	61-70	White	Cis-female	Retired, previously health evidence reviewer (at NICE) and educator	Undergraduate degree or above	Once per day	Liberal democrat
B2	22-30	White	Cis-female	Junior doctor (worked in A+E during the pandemic, currently psychiatry)	Undergraduate degree or above	Once per day	Labour
B3	22-30	White	Cis-male	Junior doctor (worked in A+E during the pandemic, currently psychiatry)	Undergraduate degree or above	Once per day	Labour
B4	22-30	White	Cis-male	Senior communications officer in an NHS trust, previous experience in pharmacy at undergraduate level	Undergraduate degree or above	Once per day	Labour
B5	41-50	White	Cis-female	Registered nurse, background in critical care, redeployed to HDU during the pandemic, agency work including on ITU	Undergraduate degree or above	Once per week	Other (Alliance party in Northern Ireland)
B6	22-30	White	Cis-female	Junior doctor (psychiatry)	Undergraduate degree or above	Once per day	Labour
B7	31-40	South Asian	Cis-female	Consultant psychiatrist	Undergraduate degree or above	Once per day	Labour

## Results

**Fig. 1:**
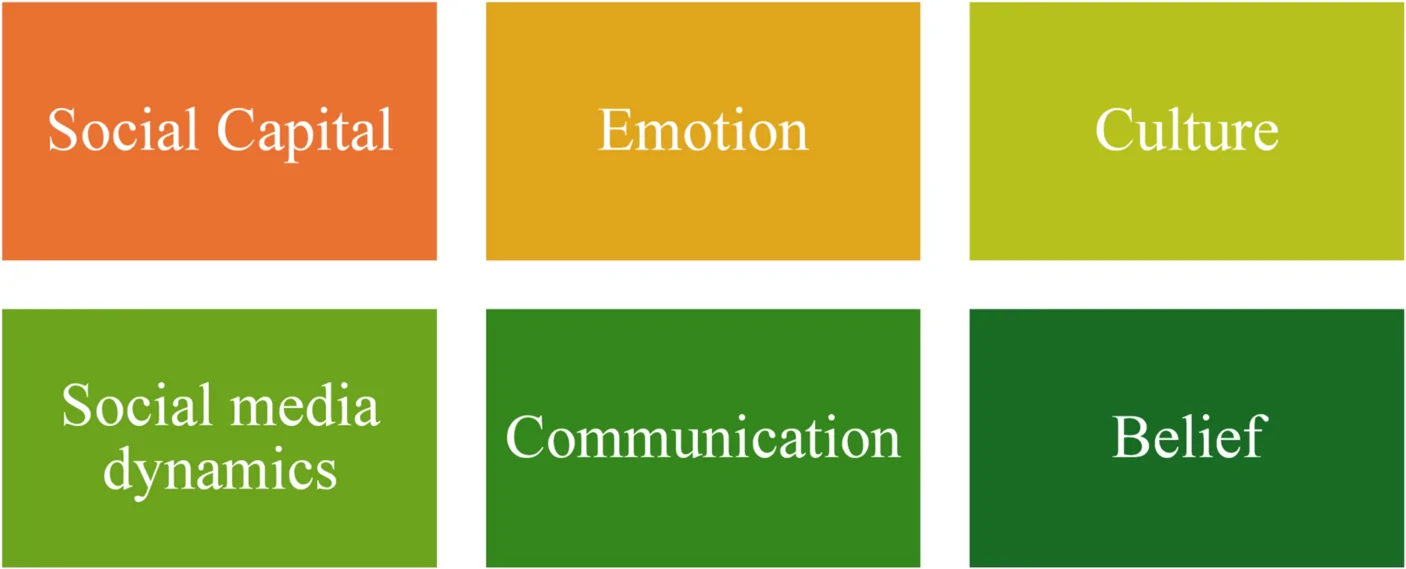
Categories of the analytical framework

Results were organised into six key categories including social capital, emotion, culture, social media dynamics, communication, and belief (Fig. 1). These categories were present in the transcripts for both groups with some similarities and contrasting points identified. This was charted in the framework matrices (Appendix 1 and 2).

### Category 1: Social Capital

Social capital is defined by Putnam as “features of social organization(sic) such as networks, norms, and social trust that facilitate coordination and cooperation for mutual benefit” [41]. In the present study participants discussed issues related to social capital which fit into three codes: Status, trust, and statistics.

Status was frequently discussed by both laypeople and professionals including status as a healthcare professional, but also status as a celebrity or influencer. Whilst participants recognised healthcare professionals had status, they suggested that this is superseded by celebrity status. Participant B2 remarked,

If…David Beckham, or Molly Mae, or Ronaldo Tweets about it then that is going to get more traction.

Indeed, most laypeople who discussed online sources of information identified celebrity journalists or content creators rather than healthcare professionals as sources that they acquired information from. For example, participant A4 discussed where he got information about masks during Covid-19,

I watch YouTube news channels and stuff… so I get some information from them” and gave examples including “secular talk and Kyle Kalinsky and Jimmy Dore.

Associated with this was another code – trust – which was something that participants often identified as influential when assessing the legitimacy of information online. Trust was identified with people who individuals have built a previous relationship with, including ‘para-social’ relationships with celebrities/influencers. Participant B5 outlined concerns about where her friends got information about mask use from,

*I know of friends who would have followed a variety of influencers whether it be about make up tips*,* house styling or something else*,* because [they] already perceived these influencers as presenting fact… they were more likely to believe that person”* … *“I think they have succeeded because they have built up this relationship of trust with their followers.*

Building trust in individuals was also cited by health professionals such as participant B1, however more often this would be with other people from a healthcare background who produced content online,

The sort of people that promote mask wearing that I trust would be Trish Greenhalgh (Professor of Primary Care Health at The University of Oxford), and people like that.

Furthermore, participants in both groups identified statistics such as likes, retweets, and follower count as giving information about the legitimacy of social media accounts talking about masks. Participant B2 lamented this phenomenon,

I think people, maybe base it on the number of likes people get means oh this must be true. And if you’ve got more followers, you must be talking the truth.

Participant A4 identified this as becoming problematic on social media leading to cultural changes in discourse online. They described “ratioing”, something which is commonplace on Twitter, where people comment “ratio” below a post with the hope of obtaining more likes than the original post [37].

Because you see now if someone has a bad tweet, you’ll see people just tweeting the word ratio, ratio, wanting to get like tons of retweets and likes, and it’s like, is that how you inform whether an opinion is good or not?

This suggests that technology is changing how people think and learn, a tenet of connectivist theory.

### Category 2: Emotion

Participants from both groups identified a range of emotions when reflecting on information being shared about masks online during the pandemic. Those that were frequently discussed were fear, anger, and burnout.

Fear was commonly discussed in relation to several issues. Firstly, fear of the pandemic itself to participants and their families. For participant A3, who spent a significant amount of time and effort Tweeting in support of masking, fear for her children’s safety was a strong motivator to try to counteract misinformation about masks online,

I’ve had children who’ve been admitted to hospital, you know my son with bronchiolitis, he was badly breathing, I’ve seen poorly kids and my daughter wasn’t well with covid at all… and that’s why I took it on.

This participant also reported that anti-mask groups were citing protecting children as their motivation for online activism and would often accuse pro-mask groups of abuse for allowing their children to wear masks,

I feel very much that they have used children, they are the most emotive subject you will ever get anybody coming out to defend. So, children have been very much used because they would provoke the biggest response.

This aligns with the care ethics theory, discussed above, that suggests people make moral decisions based on interpersonal relationships.

For healthcare professionals fear was often related to concern about receiving abuse online, and this was reported by many participants as a decisive factor in them avoiding posting about masks or trying to counteract misinformation. Participant B2 identified this as a barrier to them engaging with misinformation about masks,

I just think it’s not fair to ask people to do that in their own time, and also expose themselves to the kind of vitriol they might get, without any kind of remuneration for that. I don’t think it’s very fair.

Furthermore some, particularly junior, healthcare professionals expressed fear of lack of support from online abuse by professional bodies such as the General Medical Council (GMC), the regulatory body for doctors in the UK. This too was cited as a reason for not attempting to counteract anti-mask views. For example, participant B2 said,

I think then you get a lot of people on Twitter threaten to report people to the GMC for having different opinions to themselves.

Both layperson and healthcare professional participants reported feeling anger and observing anger from people with differing views to themselves. For healthcare professionals this was directed towards politicians who they perceived were not appropriately providing information to the public, and to anti-mask individuals/groups online. Participant B2 discussed feelings of anger when seeing her friends not doing things like masking,

I know that I had friends that were just sharing that they were doing things that they shouldn’t have done, but I would get annoyed and rant about it.

On the other hand, laypeople like participant A1 were more forgiving of other individuals online, and their anger was expressed more at the government,

I feel quite angry and confused about how they can be in positions of authority when they are so blazingly self-important and lack decency. It feels like they are not representatives of people they are representatives of themselves.

Others such as participant A5 identified anger from people when individuals tried to engage them in discussions about masks online.

But in my experience people online, or like people who believe in like anti-mask or anti-vax stuff either don’t want to fact-check or get really angry at you and say that you are silencing them.

Finally, burnout was frequently mentioned, not only from healthcare professionals who often reported that they were bringing their work home with them when they saw medical information online, but also from laypeople who said they felt overwhelmed by the sheer volume of information being shared. When asked about seeing anti-mask misinformation online participant B2 remarked,

During the pandemic with people, I’d just had enough, I didn’t want to see this when I get home, and even doctors that I might agree with but [it would] just annoy me because of the sheer volume.

A layperson, participant A2, talked about the impact on his mental health,

It’s like 24hr news isn’t it. I find it very… kind of… addictive, and I wanted to keep up to date all the time, with covid, about what was going on and I don’t think it’s the most helpful for your mental health at all.

### Category 3: Culture

The category of culture related to three focussed codes including toxic social media culture, politics, and societal culture. Participants in both groups frequently discussed the toxic social media environment, referencing abuse, pile-ons, and a general lack of motivation to learn or consider different viewpoints. Participant A3 reported being targeted by anti-mask accounts,

I found out that they’d actually found other sources of my social media and copied pictures of my children, at which point I decided to create an anonymous Twitter account where…they couldn’t target me personally.

Similarly, participant B4 discussed anti-mask accounts trying to create pile-ons against pro-mask accounts online,

They are trying to get other people involved, and trying to create what we call a pile on, so they want more people to get involved and then it spirals and spirals.

Participants in both groups also frequently mentioned politics. However, rather than the right versus left political axis, overwhelmingly they identified anti-authoritarian views as influencing anti-mask beliefs. For example, participant B3 remarked,

A lot of it came down to masks might work but my freedom is more important.

Furthermore, several participants specifically identified that masks can be used as a powerful symbol of an individual’s anti-authoritarian beliefs. For instance, participant A1 observed,

I think like it’s just quite a simple symbol. Like you can be anti-vax and you wouldn’t know, or you can be pro-vaccine and you wouldn’t know… but a mask is something very symbolic, and you can show that you are resisting.

Finally, participants from both groups also discussed the societal culture of the UK. Participant A4 discussed differences between the UK and Asian countries that had experienced SARS in the past,

After SARS … it became quite common to wear a face mask, but you see here it feels like no one ever really wants to see them again now… what are we expecting to happen next time a pandemic comes around… it seems like now we haven’t really learnt much… like it was just some social experiment.

Furthermore, there were references to a culture within the UK of suspicion or lack of trust in experts/professionals. As discussed, this can be seen as a kind of epistemic injustice. For example, participant A4 stated,

We do live in a horrible world where people don’t actually trust that many people anymore, … even journalists, people don’t trust their opinions anymore, politicians people don’t trust their opinions anymore.

### Category 4: Social Media Dynamics

Perhaps surprisingly, many participants from both groups were aware of social media dynamics that lead to misinformation being spread online, with many making explicit references to both polarisation and echo chambers. Again, this suggests that social media technology is changing how we think and learn. Participant A4 highlighted the issue of echo chambers,

You can so easily get sucked into an echo chamber, so I’ve always been conscious of that even trying to follow different accounts…even from things that I would like disagree with.

Similarly participant A5 discussed polarisation,

So, it becomes like really polarised, and it becomes difficult to engage people and have conversations around it because they can just tell you that they found one person that said a different thing.

Many participants, both laypeople and healthcare professionals, referenced the sheer amount of information online being overwhelming i.e., information overload. Participant A1 acknowledged experiencing this phenomenon,

I would like to say that each time I went and investigated, researched and did all that sort of thing. But I think there’s an extent to which I probably saw the mass of information, and got the information from people I respected.

### Category 5: Communication

Another category was communication; including the codes style of communication and managing cognitive load that resulted from the volume of information online.

Participants from a healthcare profession background often referenced that social media required a completely different style of communication from clinical practice or teaching, and that they did not feel prepared for this. Differences that were identified included social media being a public forum, people online not being motivated to learn, and only having one opportunity to engage with an individual online rather than being able to have multiple interactions with people in clinical practice and teaching. For example, participant B6 stated,

I think it’s very different, it’s a very different platform, the whole structure of the way you communicate with people is different.

On the other hand, layperson participants often reported feeling patronised due to a didactic style of communication being used by healthcare professionals when talking about masks online. Participant A1 posited,

You need to engage with people, people need to feel like they are benefitting from it, it can’t feel like you are being talked down to, you are being excluded.

Secondly, laypeople discussed several ways which made it easier for them to understand information about masks being shared online, which helped to manage their cognitive load. Participant A2 discussed the importance of infographics,

I think there were a few sort of very good infographics coming out of certain places, which I do think if you can make something that… is actually science led…but that actually I can post on Instagram and people who have no science background can read that and understand it I think that is very helpful.

### Category 6: Belief

The final category was belief, where participants identified that anti-mask views were often associated with other beliefs, and that once people made their minds up beliefs were fixed and difficult to change.

Participants from both groups identified anti-mask views being associated with other beliefs such as specific political beliefs, or other health behaviours such as vaccination. Participant A3 felt that anti-mask accounts were using anti-mask views as a platform to move onto anti-vaccine beliefs,

I started saying in September this lot aren’t just against NPIs (non-pharmaceutical interventions) … this lot are building to something, [like the] anti-vax movement. If they undermine everything before vaccinations, if they’ve seeded that thought of doubt in people’s minds that things aren’t working, and then they move on to things like that.

Furthermore, participant A5, who identified their gender as non-binary, reported they had often seen anti-mask messages associated with transphobia online.

I’ve seen people who post like negative things around trans people, if I look at their profiles, they often are also posting anti-mask stuff.

Finally, participants in both groups often identified that once beliefs are formed, they can be fixed and difficult to change. For example, participant B3 admitted to experiencing this themselves,

But I was going into reading the tweets knowing that what I thought [about masks] was correct, and therefore I wouldn’t change my mind.

## Discussion

Through 12 in-depth interviews, six key categories relating to misinformation around mask wearing during the pandemic were revealed: Social capital, emotion, culture, social media dynamics, communication, and belief.

### Social Capital

Social capital was seen by participants from both groups as a means through which to influence discourse online. In social media theory, online social capital is seen as distinct from offline social capital because of the wide range of people that can be reached instantly [42]. Social capital can be achieved through perceived status such as professional standing via titles e.g., doctor, or through celebrity status, or trust (through long-standing social media relationships), and through broad influence such as generating likes and retweets (i.e., statistical ‘weight’). Although participants recognised their role as healthcare professionals provided an element of status, they reported that this was superseded by celebrity status, and this was a major reason why healthcare professionals lacked motivation to engage in counteracting misinformation about masks online. As discussed, this could be interpreted as a form of epistemic injustice which likely contributes to this lack of motivation. Other reasons included lack of time, lack of skills for communicating in this medium, and perceived lack of support from professional organisations.

Participants also identified that influencers or celebrities online gain social capital by building trust with social media users in ‘parasocial’ relationships which then leads to them being seen as trustworthy when discussing issues such as mask use. This is similar to findings by Lockyer et al. [30] discussed earlier. In our study this was also true for healthcare professionals, but their trust was placed more with other healthcare professionals online. This may suggest that education including in healthcare related fields is not protective against the effects of misinformation. Rather, what may be more influential is being part of the ‘in-group’ that is more likely to demonstrate predominantly pro-mask beliefs. This corresponds to the connectivist view that suggests nurturing and maintaining connections is important in facilitating continual learning.

Furthermore, social capital includes statistics such as likes, retweets, and followers. In our study participants identified that these statistics are used to assign legitimacy to accounts or posts online. One interpretation of the relevance of social capital to misinformation relates to Marxist theory, where modern Marxist theorists have argued that in the era of mass information, knowledge has been commodified and there is a new form of “immaterial labour” [29]. Indeed, influencers can now make a living from creating ‘content’ online. In this environment, which it can be argued is oversaturated with information, social capital has a semiotic function which signifies to readers that their content has value and therefore draws attention. Marx himself outlined the labour theory of value which suggests the value of a commodity can be measured by the number of labour hours required to produce it [45]. Arguably, this is equally true for the production of knowledge i.e. the opinion of a virologist on Covid-19 has value because of the years of labour time they have spent to understand the dynamics of viral transmission. Traditionally titles such as “Professor” and “Doctor” have been used as proxies to identify how much epistemic labour time has been spent to understand a subject. However, in the online environment where anyone can call themselves any title this credibility is potentially lost, and people may seek information from less reputable sources.

On social media individuals form into clusters, or groups of users with similar online activities or connections. Johnson et al. report that anti-vaccination clusters offered a wider range of potentially attractive narratives and that pro-vaccine clusters were far more monothematic leading to anti-vaccination clusters being more intertwined with undecided clusters than pro-vaccination clusters were [26]. Thus, suggesting that undecided clusters are connecting more with misinformation than reputable information. Researchers in other areas have shown that communities of practice can form around a variety of interests [18, 32, 36, 52]. For example, Waldron, in a study of an online music community reports that content creators can engender formation of learning communities, suggesting that YouTube videos can be vehicles of agency through which to promote and engage participatory culture [52]. Therefore, if healthcare professionals from diverse backgrounds build social capital and become influencers online, it is possible that what Johnson et al. describe could be counteracted by generating diverse online communities that could act as communities of practice. This idea may also be supported by evidence from Dennis et al. who showed messages tailored to people’s identity were more likely to increase compliance with masking and lockdowns [13]. However, the rise of influencers has some clear ethical issues which must be addressed, not least the potential of corruption through paid advertisements and sponsorships.

### Emotion

Participants described negative emotional responses including fear, anger, and burnout. Fear included fear of the pandemic itself but, importantly, also fear of abuse when attempting to counteract misinformation online. Related to this was concern, particularly from junior doctors, that they would be referred to the GMC for their comments and not supported by them. These concerns were another reason cited by participants for not engaging with misinformation. A similar finding was reported by Bautista et al. [4].

A further reason for healthcare professionals not engaging with misinformation online was burnout. Healthcare professional participants identified the feeling that they were bringing work home with them when they read medical information about Covid-19 on social media. Even prior to the pandemic prevalence of burnout was already reported to be high [25]. Recent evidence in the field of cognitive load has suggested that emotion can restrict the capacity of working memory, competing with task relevant processes [49]. Therefore, both fear and burnout secondary to online social media discourse about the pandemic is likely to have deleterious effects on healthcare professionals’ capacity to learn about and counteract misinformation online. For laypeople in our study burnout was related to receiving constant news regarding Covid-19 with many reporting that they consciously took a step back from social media.

Fear as a means through which to influence others was identified by one of our participants, who described anti-mask and anti-vaccine groups exploiting fear about the wellbeing of children during the pandemic, this is in line with data from Hughes et al. [24] outlined above. On the other hand, fear of harm to her children was a strong motivator for a participant in our study to spend a significant amount of time trying to counteract misinformation about masks online, aligning with the theory of care ethics.

### Culture

Toxic social media culture, politics, and societal culture were all issues that participants identified during the interviews for not engaging in discussions online. Part of this was a belief from participants that in the online environment there is not a culture of willingness to learn.

Like other studies, our participants felt that politics was relevant to the issue of misinformation during Covid-19. However, unlike most other studies the main concern participants had witnessed in online discourse related to masks was anti-authoritarianism rather than a clear right-left political divide. This is in-line with data from Al-Ramahi et al. who showed that one of the most common themes of anti-mask Tweets during the Covid-19 pandemic was constitutional rights and freedom of choice [1].

Government measures such as the police, crime, sentencing and courts bill enacted in the UK in 2022, which introduced a range of new police anti-protest powers are highly likely to create resistance from certain groups. This combined with apparently authoritarian Covid-19 policies such as jail sentences and large fines for breaking lockdown rules, alongside apparent disregard for these policies by senior political leaders, are likely to reduce trust in the government and associated institutions, including the NHS, amongst those groups. Masks have now been associated with these authoritarian measures, and a strong anti-mask culture has grown within the UK. Crucially, as described by Chen et al. memory of a previous pandemic can lead to better compliance with containment measures in future pandemics [8]. If masking continues to be associated with authoritarianism in the UK this could, therefore, have disastrous consequences if there are new variants of Covid-19, or in future pandemics. The question that must be answered then is how can masks be separated from the idea of oppression?

Friere, in his theoretical political work argues that liberation of the oppressed (working class masses) can only occur through dialogue rather than didactic methods: “Propaganda, management and manipulation – all arms of domination - cannot be instruments of their rehumanisation. The only instrument is a rehumanising pedagogy in which the revolutionary leadership establishes a permanent relationship of dialogue with the oppressed” (Friere, 1970/2017). Therefore, it may be argued that for healthcare professionals to effectively support the public in protecting themselves from communicable diseases a permanent relationship of dialogue should be established. Social media is an effective means through which this could be achieved that can facilitate direct engagement between healthcare professionals and members of the public. This could also be of benefit in other conditions, for example the UK has recently seen a high-profile case, discussed earlier, where a young person died after rejecting chemotherapy treatment based on misinformation shared by her mother [46, 47].

### Social Media Dynamics

Connectivism builds on the idea that technology is redefining and shaping how we think and learn. Participants in our study recognised negative consequences of social media dynamics including echo-chambers, polarisation, and information overload. Polarisation was felt to be a key issue during the pandemic, and often participants identified that this increased over time. In a study using simulations of social networks pre-Covid, Garibay et al. found the effect of polarised posts was accumulation of a subset of the network that leads to an increased success in their ability to propagate content through intermediaries [19]. This may be a reason for which polarisation may increase over time, and also may partially explain evidence from other studies suggesting that questionable information spreads faster than reliable information during the Covid-19 pandemic [10, 33].

Our participants described being overwhelmed by the sheer amount of information about masks during the pandemic and described not having the energy to fully engage with all the information, which can be described as cognitive overload. This was also true for healthcare professionals in the current study. The relevance of cognitive load has been reported by three other studies [28, 31, 38]. As noted earlier, celebrities and influencers have social capital and as role models can help in management of cognitive load, even though they lack knowledge on the issue.

### Communication

Style of communication, and methods of communicating knowledge helped participants process information or manage cognitive load. Layperson participants referenced strategies such as infographics which helped them to understand the science behind mask use, which is supported by other studies within the literature [14, 51]. Notably, healthcare professionals outlined that there was a significant difference between communicating online and in their practice, both pedagogical and clinical. Some laypeople too, suggested that healthcare professionals were not adept at communicating in the social media environment. This shows that for healthcare professionals to be successful at counteracting medical fake news or misinformation online there should be a focus on this from a medical education perspective. Indeed, some have called for this to be added to medical school curriculums, for example Minnesota department of medicine [34]. Layperson participants in our study described that there is often a tone of superiority, arrogance, and a didactic style of communication which they did not appreciate. This could support the idea of Friere, outlined earlier, that a dialogue should be created rather than dictating to or managing laypeople.

### Belief

This final category included that anti-mask views were associated with other beliefs and that once beliefs had been formed, they were difficult to change. That belief in Covid-19 misinformation was associated with other beliefs is consistent with the literature. Others have shown that belief in different conspiracies was associated with belief in non-reputable information about Covid-19. For example, Georgiou et al. demonstrated belief in Covid-19 conspiracies was associated with other conspiracy beliefs such as evidence of alien contact being concealed from the public, amongst others [20]. The timing of feedback to counter misinformation is important [23] and Brashier et al. recently reported the effects of timing of feedback when correcting political misinformation online [6]. They found fact-checking that appeared immediately after exposure to misinformation was more effective at increasing belief in true headlines than presenting corrections to misinformation during or before exposure [6]. This suggests any counter to misinformation should occur early on after the misinformation begins to spread. This may be supported by data from Cui & Kertész who suggested that public attention was most focussed early in the pandemic [12]. Therefore, it is likely that, after not receiving feedback on beliefs about masks at an appropriate time, beliefs become entrenched and difficult to change. This suggests that any healthcare professionals attempting to counteract medical fake news in future should act rapidly, or as Dr Michael Ryan of the WHO stated about Covid-19 itself “the greatest error is not to move” and “speed trumps perfection” [54].

### Limitations of this Study

It is important to note some limitations to this study. Firstly, although some participants in the study were not strongly pro-masking, there was no participant who expressed significant anti-mask views. Similarly, all participants in the study reported they had voted for a left-wing or centre-ground political party in the 2019 UK general election, and therefore overtly right-wing views are not represented in the study. This may be secondary to the method of recruitment. However, another contributing factor could be that people with anti-mask views may have lost trust in institutions, such as universities and ‘experts’, and therefore did not agree to take part. The authors acknowledge their professional backgrounds as healthcare educators may have influenced interpretation, and reflexive memos were used to mitigate this.

## Conclusions

This study highlights the profound challenges that healthcare professionals face when attempting to engage with and counteract health-related misinformation online. Viewing it through the lens of connectivism, misinformation can be seen as a networked learning phenomenon shaped by connections, trust and technology, rather than individual ignorance.

Despite the proliferation of misleading content during the Covid-19 pandemic, particularly concerning mask usage, most healthcare professionals interviewed reported disengagement from online discourse due to fear of abuse, lack of institutional support, and inadequate training in digital communication. Simultaneously, lay participants expressed frustration at condescending communication styles and perceived healthcare messaging as didactic or exclusionary.

These findings have significant implications for policy and practice. Firstly, there is a clear need for targeted digital communication training within healthcare education. This training should not only equip professionals with the skills to engage effectively and respectfully online but also help them understand the dynamics of digital trust, social capital, and emotion-laden discourse that characterise social media environments. Medical schools and postgraduate training programmes should formally incorporate curricula on digital health communication, focusing on how to build trust, use accessible language, and manage the cognitive load of diverse audiences.

Secondly, healthcare regulators and professional bodies must take an active role in providing support and protection for professionals who responsibly counter misinformation online. Concerns about institutional reprisal, such as fears of GMC complaints, served as a significant deterrent in this study. Clear guidance, supportive policies, and rapid institutional responses to online abuse could empower more professionals to engage constructively and confidently in the public sphere.

Thirdly, the study underscores the importance of early, identity-sensitive, and dialogic responses to misinformation. Public health messaging should be pre-emptive, appear early in misinformation cycles, and come from diverse, relatable messengers. Engaging influencers from different backgrounds to act as trusted intermediaries may enhance the reach and acceptance of health messages, especially among groups that are sceptical of traditional authority figures.

Moreover, tech platforms and policymakers must work collaboratively to strengthen digital environments. Algorithms that prioritise emotionally charged or polarised content amplify misinformation. Regulatory frameworks should therefore promote transparency, elevate evidence-based content, and incentivise platforms to prioritise accuracy and harm reduction. Simultaneously, public-facing digital literacy initiatives, particularly those targeting vulnerable or disengaged populations, are crucial to foster critical evaluation of online information.

Finally, if beliefs are socially and emotionally reinforced and difficult to shift once formed, future responses to public health crises must avoid alienating sceptical populations. This requires long-term, trust-building engagement, not just crisis communication. Social media offers the potential for participatory, bidirectional dialogue between professionals and the public. Leveraging this potential could transform the role of healthcare professionals from authoritative broadcasters to facilitators of informed public discourse.

In sum, countering health misinformation effectively demands a coordinated, system-wide response that integrates education, institutional reform, platform accountability, and public engagement strategies. Without such multifaceted action, we risk entering future public health emergencies no better equipped than we were during Covid-19.

## Data Availability

No datasets were generated or analysed during the current study.

## Appendix 1

See Table 4.

**Table 4:** Framework matrix for group A (laypeople)

Case	Social capital	Emotion	Culture	Social media dynamics	Communication	Belief
A1	• Trusting people who they have formed a previous relationship with online (positive/negative) – people who they didn’t like weren’t wearing masks, people who they like were. • Don’t follow any doctors really – only through left wing people they follow that they got exposure to healthcare professionals	• Talks about motivation to protect family • Masks affecting social interaction and makes them not want to wear them • Anger towards politicans – but understanding towards laypeople • Feeling overwhelmed	• The political climate of the UK with a lot of anti-authoritarian/libertarian thought • Masks as a symbol of political beliefs • Talks about masks being a “culture war” issue • Social pressures – feeling embarrassed to wear a mask when others weren’t	• Talks about a dichotomy of views on mask wearing, and there being a big split. • Discusses overwhelming amounts of information – and uses people he trusts on social media to deal with this • Specifically references echo chambers and trying to avoid that by following other people • Reduced use of social media feeling overwhelmed	• Talks about communication online being different and not feeling healthcare professional would be particularly prepared for that and be able to “market themselves” online.	• Talks about beliefs on mask use being related to previously held political beliefs
A2	• Also talks about getting their information from trusted sources – including some doctors, organisations, but also influencers online again. • References social media numbers guiding who to trust. • Using people who they previously agreed with as a guide	• Anger at people spreading misinformation • Also feeling overwhelmed with amount of information. • Anger at anti-mask doctors. • Talked about motivation to protect others rather than themselves driving their mask use	• References libertarian culture again • References “culture war” • Talks about a general lack of trust in professionals or experts in the UK	• References their Twitter being a bit of an echo chamber, only following certain accounts, describes as social media “bubble”. • Also using social media less because of information overload	• Saw people ridiculing people who didn’t wear masks online and didn’t think this was a helpful tactic • Felt communicating points using infographics was helpful. • Felt making the main takeaways of scientific evidence clear on social media was a good way of communicating it – talked about infographics that can be shared on Instagram	• People who are anti-mask are often “anti” a lot of things • Talks about people “sticking to their beliefs” and being very fixed
A3	• Talks about people getting misinformation from sources they’ve trusted in the past – journalists such as alison pearson, GB news, talks about a layperson who is anti-mask/anti-vax being given a weekly column in the telegraph • Talks about these groups building relationships with people over a period of time • Talks about “bad” doctors that are used to spread misinformation	• Strongly motivated to protect others, sick neighbour and children – leading to her spending significant amounts of time trying to counteract misinformation herself • Talks about people exploiting emotion with regard to children to spread misinformation • Lots of anger towards people spreading m misinformation. • Anger towards specific medical speciality	• Culture online is very toxic – she received lots of personal abuse, pile-ons etc. had to create another social media account without any personal information	• Talks about views being split on social media with lots of groups with the opposite view to them	• Liked things explained in an analogy – e.g. the Swiss cheese model	• Strong belief that the main anti-mask sentiment is from long-term anti-vaxxers and comes from anti-vax pressure groups. • Talks about beliefs being very fixed once formed – doesn’t think she has changed anyone’s mind just the undecided people.
A4	• They talk about their own trusted sources of information that they have built a relationship with before. • References to people only caring about likes and re-tweets online – “ratio-ing” • Talks about para-social relationships – building relationships with people online for years.	• Talks about anger at misinformation and at some of the way that pro-mask groups communicate. • Talks about feeling exhausted.	• Feels that the issue got politicised quickly. • Talks about difference between eastern and western cultures. • Talks about the influence of the US	• Feels the social media environment is not conducive to real discussion. • Talks about echo chambers.	• Talks about people on the pro-mask side virtue signalling, feels like they avoided actually talking about the science and would have appreciated this. • Talks about bodies changing their messaging. • References people talking down to people, calling them idiots etc.	• Talks about previously held beliefs - politics
A5	• Talked about their views being influenced by people who they trust • Follows accounts who they agree with politically • Looks at who follows people	• They also talk about motivation to protect others more than themselves • Anxious from all the negative news • Anger at misinformation	• References an “anti-institutional” culture	• Felt overwhelmed by social media and has been using it less • Feeling like the social media environment is oversaturated with information	• Talks about adapting communication style with simple language and diagrams. • Would like healthcare professionals communicating online who reflect their identity	• Talks about the association with other beliefs – in particular transphobia

## Appendix 2

See Table 5.

**Table 5:** Framework matrix for group B (healthcare professionals)

Case	Social capital	Emotion	Culture	Social media dynamics	Communication	Belief
B1	• Has people that she trusts too – however these are medical people e.g. professor Trish Greenalgh • Talks about people forming previous relationships with celebrities • They have noticed a more positive response from people they have a previous relationship with	• Gets very angry at misinformation • Feels defeated and loses motivation to try and counteract fake news	• Talks about people being anti-authoritarian/libertarian • Culture of not trusting experts – talks about people with history degrees spreading misinformation • Says they don’t think as a country we are good at critical thinking • Twitter culture divisive, angry and aggressive – toxic • Talks about being personally attacked for trying to counteract misinformation	• Talks about moderating their own social media environment – i.e. echo chambers • Lack of accountability on twitter – people just delete tweets later without stating why or that they were wrong	• Talked about Twitter/social media being a different environment to communicate in • Tried nudge technique • Talks about having to convey a clear message in a few characters	• Talks about beliefs being very fixed, not seen anyone change their mind • Beliefs get more and more entrenched over time
B2	• Talks about people forming relationships with celebrities such as David Beckham and Molly Mae • Talks about anti-mask doctors being used to spread misinformation • Talks about status being important • Talks about number of likes and follower counts etc.	• Felt burnout, working in A + E seeing covid and didn’t want to come home and see a lot of anti-mask misinformation • Fear of reprisal from the GMC if they did try and engage online – people making threats of GMC referral • Anger at misinformation	• Talks about people seeming worried about authoritarianism • Talks about a punitive culture from medical bodies that prevent them from engaging online. • Talks about UK culture being different to other places where more people wear masks. • Doctors associated with the government/authoritarianism	• Also talks about moderating their online environment – people with similar political views • Even talks about unfollowing people with different views about masks • Spent less time on twitter	• Saw other doctors communicating in a “holier than thou” way which they didn’t think was helpful • Talked about lack of clarity in messaging	• Makes reference to people having firmly held beliefs that are difficult to change
B3	• Trusts people in positions of authority that he respects • Talks about people only believing “facts” from sources they trust • Talks about the blue tick	• Lack of confidence in counteracting misinformation	• Talks about influence from America – anti mask posts coming from America • Talks about a culture of people not believing in facts anymore.	• Talked about his Twitter feed being very selected • Talks about social media being very polarised	• Twitter is a very different place to communicate in – can’t gauge people’s reactions, its public and can be seen by anyone • Feels doctors acknowledge uncertainty too much and need to be clearer • Importance of using simple language • Using humour • Don’t think they previous experience is helpful in communicating online • Seen people ridiculing others.	• Views being fixed – talks about knowing he can’t change people minds • Talks about anti-mask views being associated with other beliefs e.g. gun ownership, being a republican in America.
B4	• Feels people have been influenced by celebrities such as Lawrence fox • Feels his efforts were possibly more effective on his personal account because they were people who know and trust him	• Burnout from social media use at work so used it less personally at home • Has to hold himself back from getting angry	• Culture of mistrust in organisations and corporations • People linking the NHS with the government • Masks as a symbol of rebellion • Toxic social media culture - people trying to create pile-ons online to people they don’t agree with	• Talked about things being very polarised online. • References echo chambers • Talked about engagement dropping over time	• Talks about the importance of choosing who to engage with – so not to give them more attention “don’t feed the troll” • Talks about the importance of actually engaging with people rather than putting out didactic statements • Feels people might respond better to less senior people communicating online – e.g. nurses rather than Chris Whitty as they are more relatable • Talks about directing people to evidence so they can read it	• People having fixed beliefs – talked about it being a “fight for the middle ground”
B5	• They got their information from governing bodies such as the WHO • Thinks a lot of people got their information from influencers • Influencers have built a previous relationship with people • Talks about numbers of followers	• Refrained from using social media as “self-preservation” • Burnout and didn’t want to take work home with her • Anger at misinformation	• Talks about a toxic environment online with trolls	• Unfollowed anyone she disagreed with – creating an echo chamber	• Talked about deciding who to try to convince and who not to • Feels people will listen to their peers more than “someone in a suit and tie” on the news • Feels its important for people providing information to be relatable and for the public to have people that “look like them” • Non-confrontational style of communication not saying, “you’re wrong”. • Feels communication/teaching online very different to normal practice – can’t have as much of a discussion	• Talks about some people being very set in their views
B6	• Followed other professionals talking about covid online • Both sides referring to “pet experts”	• Anger at people ridiculing people for wearing masks online • Burnout- felt that engaging with people online would be more work which they aren’t paid for • Doesn’t feel protected from abuse online as a junior doctor	• Talks about culture of mistrust for “people who are knowledgeable about these things” • Talks about the difference between the UK and New Zealand – more of an agreement with the government in New Zealand • Talks about influence from the US • Libertarian viewpoint • Feeling of disenfranchisement and that doctors are part of the establishment • Toxic environment with lots of vitriol online		• Feels taking the approach of ridiculing people makes people stick to their position more • Comes better from someone who is aligned with the person in other ways – feels we should recruit from diverse populations • Talks about being clear and confident in messaging about masks working • Doesn’t feel prepared for communicating in the online environment from previous experience	• Talks about people’s views becoming more entrenched over time
B7	• Gets their information from organisations such as the royal college	• Irritated by misinformation	• People talking about freedoms and “human rights” with regard to mask wearing • Being “anti-mask” becomes part of their identity • Talked about being trolled	• Twitter positively reinforces your own views • Talked about polarisation	• Not room for any constructive conversation online • Social media environment is very different – people aren’t open to learning, you need to have repeated messages from different people • Don’t be confrontational	• Talked about how it doesn’t seem possible to change anyone’s mind
